# Supplementary material for: Offspring chemical control of adult reproductive transitions in a social insect
Source: Proc Natl Acad Sci U S A. 2026 Apr 8;123(15):e2526776123. doi: 10.1073/pnas.2526776123 (PMC13079936; doi:10.1073/pnas.2526776123)
Supplement: Supplementary file 5 — Dataset S04 (PDF) [file pnas.2526776123.sd04.pdf]

```
#library
library(ggplot2)
library(dplyr)
library(DHARMa)
library(ggpubr)
library(dunn.test)


#Figure 1-------------------------------------------------------------------------------------
#data------------------------
fig1=read.table("Dataset S1.txt", header=T)

fig1$batch <- as.factor(fig1$batch)
fig1$treatment <- as.factor(fig1$treatment)
fig1$eggs <- as.numeric(fig1$eggs)
fig1$ants <- as.numeric(fig1$ants)

# last experimental day
fig1_lastday=subset(fig1, day=="8")

# remove arenas where >33% (1/3) of the ants died
fig1_lastday_filtered=subset(fig1_lastday, ants > 7 ) #nests with 8 or more ants are kept

# sample sizes
rep_counts_fig1 = fig1_lastday_filtered %>%
  group_by(treatment) %>%
  summarise(n = n())
rep_counts_fig1

# median and s.d. eggs per ant on last experimental day
tapply(fig1_lastday$eggsperant, fig1_lastday$treatment, median)
tapply(fig1_lastday$eggsperant, fig1_lastday$treatment, summary)


# Does treatment affect egg laying?--------------
kruskal.test(eggsperant ~ treatment, data = fig1_lastday_filtered)
dunn.test(fig1_lastday_filtered$eggsperant, fig1_lastday_filtered$treatment, method = "BH",
list =T, altp = T)


#date of first egg laying------------------
# For each replicate, find the first day where egg number > 0
fig1_replicate_removed = setdiff(fig1_lastday$ID, fig1_lastday_filtered$ID)#show ID of
replicate removed
fig1_replicate_removed #--> none were removed

firsteggf1 <- fig1%>%
  group_by(ID) %>%  # Group by the replicate ID
  filter(eggs > 0) %>% #remove colony that did not lay eggs
  arrange(ID, day) %>%  # Sort by ID and day
  slice(which.max(eggs > 0)) %>%  # Find the first day where eggs > 0
  select(ID, day, eggs, treatment, batch)

# median and s.d. of latency to lay eggs per treatment
tapply(firsteggf1$day, firsteggf1$treatment, median)
tapply(firsteggf1$day, firsteggf1$treatment, summary)

#Figure 3 ------------------------------------------------------------------------------

#data------------------------
fig3=read.table("Dataset S3.txt", header=T)

fig3$batch <- as.factor(fig3$batch)
fig3$treatment <- as.factor(fig3$treatment)
fig3$eggs <- as.numeric(fig3$eggs)
fig3$ants <- as.numeric(fig3$ants)

fig3_lastday=subset(fig3, day=="9")
```

```
# remove arenas where >33% (1/3) of the ants died
fig3_lastday_filtered=subset(fig3_lastday, ants > 7 )
fig3_replicate_removed = setdiff(fig3_lastday$ID, fig3_lastday_filtered$ID)#show ID of
replicate removed
fig3_replicate_removed

# sample sizes
rep_counts_fig3 = fig3_lastday_filtered %>%
  group_by(treatment) %>%
  summarise(n = n())
rep_counts_fig3

# Does treatment affect egg laying?--------------
# remove "larvae contact" treatment from analyses as it is here as a sanity check with low
replicate number
fig3_selection=subset(fig3_lastday_filtered, treatment!="Larvaecontact_pupae")

kruskal.test(eggsperant ~ treatment, data = fig3_selection)
dunn.test(fig3_selection$eggsperant, fig3_selection$treatment, method = "BH", list = T, altp =
T)


# median and s.d. eggs per ant per treatment on last experimental day
tapply(fig3_selection$eggsperant, fig3_selection$treatment, median)
tapply(fig3_selection$eggsperant, fig3_selection$treatment, summary)

# Latency to lay eggs as a function of treatment-------------------------
# For each replicate, find the first day where egg number > 0

fig3_replicate_removed = setdiff(fig3_lastday$ID, fig3_lastday_filtered$ID)#show ID of
replicate removed
fig3_filtered=subset(fig3, ID!="MEHMP_pupae_1" & ID!="pupae_15") # remove replicates where >33%
of the ants died the last day

firsteggf3 <- fig3_filtered %>%
  group_by(ID) %>%  # Group by the replicate ID
  filter(eggs > 0) %>% #remove colony that did not lay eggs
  arrange(ID, day) %>%  # Sort by ID and day
  slice(which.max(eggs > 0)) %>%  # Find the first day where eggs > 0
  select(ID, day, eggs, treatment, batch)

kruskal.test(day ~ treatment, data = firsteggf3)

# median and s.d. of latency to lay eggs per treatment
tapply(firsteggf3$day, firsteggf3$treatment, median)
tapply(firsteggf3$day, firsteggf3$treatment, sd)
```
