# Supplementary material for: Offspring chemical control of adult reproductive transitions in a social insect
Source: Proc Natl Acad Sci U S A. 2026 Apr 8;123(15):e2526776123. doi: 10.1073/pnas.2526776123 (PMC13079936; doi:10.1073/pnas.2526776123)
Supplement: Supplementary file 6 — Dataset S05 (PDF) [file pnas.2526776123.sd05.pdf]

```
#library
library(ggplot2)
library(dplyr)
library(ggpubr)


#Figure 1------------------------------------------------------------------------
#data
fig1=read.table("Dataset S1.txt", header=T, dec=".")


fig1$batch <- as.factor(fig1$batch)
fig1$treatment <- as.factor(fig1$treatment)
fig1$eggs <- as.numeric(fig1$eggs)
fig1$ants <- as.numeric(fig1$ants)

custom_order_fig1 <- c("larvae_contact",
                   "larvae_distance",
                   "pupae")

fig1$group <- factor(fig1$treatment, levels = custom_order_fig1)
fig1_lastday=subset(fig1, day=="8")

fig1_lastday$treatment <- factor(fig1_lastday$treatment, levels = c("larvae_contact",
                                                    "larvae_distance",
                                                    "pupae"))

# count replicates per treatment
rep_counts_fig1 = fig1_lastday %>%
  group_by(treatment) %>%
  summarise(n = n())


#graph last day Figure 1
#median + IQR
ggplot(fig1_lastday, aes(x = treatment, y = eggsperant)) +
  geom_jitter(aes(fill = treatment, color = "black"),  # Fill by treatment, black outline
              height = 0, width = 0.1, size = 5, shape = 21, stroke = 0.5) +  # stroke controls
outline thickness
  stat_summary(
    fun.data = function(x) {
      q <- quantile(x, probs = c(0.25, 0.75))  # Calculate Q1 and Q3
      m <- median(x)  # Use median instead of mean for consistency with IQR
      data.frame(y = m, ymin = q[1], ymax = q[2])  # IQR as error bars
    },
    geom = "errorbar",
    width = 0,
    color = "black",
    size = 0.7
  ) +
  stat_summary(
    fun = median,  # Use median instead of mean for consistency with IQR
    geom = "point",
    shape = 18,
    size = 5,
    color = "black",
    fill = "white"
  ) +
  xlab("") +
  ylab("Number of eggs per ant") +
  theme(
    legend.position = "none",
    axis.title.x = element_text(size = 20),
    axis.text.x = element_text(size = 20, angle = 0, hjust = 0.5),
    axis.text.y = element_text(size = 18, angle = 0, hjust = 0.5),
    axis.title.y = element_text(size = 26),
    plot.title = element_text(hjust = 0.5, size = 25),
    panel.background = element_rect(fill = "white", color = NA),  # no square border
    panel.grid.major = element_blank(),
```

```
    panel.grid.minor = element_blank(),
    plot.background = element_rect(fill = "white", color = NA),
    axis.line = element_line(color = "black", size = 0.5),  # keep axis lines
    plot.margin = margin(10, 10, 10, 10),
    panel.spacing = unit(0, "lines")
  ) +
  scale_y_continuous(limits = c(-0.1, 1.55), breaks = seq(0, 1.4, by = 0.2)) +
  scale_fill_manual(values = c(
    "larvae_contact" = "#D7191C",
    "larvae_distance" = "#2E8A47",
    "pupae" = "#2C7BB6"
  )) +
  scale_color_manual(values = c(
    "black" = "black"  # Explicitly set outline color to black
  )) +
  annotate("text", x = 1, y = 0.2, label = "", color = "black", size = 8, fontface = "bold") +
  annotate("text", x = 2, y = 1.0, label = "", color = "black", size = 8, fontface = "bold") +
  annotate("text", x = 3, y = 1.55, label = "", color = "black", size = 8, fontface = "bold") +
  scale_x_discrete(labels = c(
    "larvae_contact" = "Larvae
    contact",
    "larvae_distance" = "Larvae
    distance",
    "pupae" = "No
    larvae"
  )) +
  geom_text(data = rep_counts_fig1, aes(x = treatment, y = -0.1, label = paste0("n = ", n)),
            inherit.aes = FALSE, size = 6)+
  annotate("text", x = 1, y = 0.2, label = "a", color = "black", size = 5, fontface = "bold")+
  annotate("text", x = 2, y = 0.9, label = "b", color = "black", size = 5, fontface = "bold")+
  annotate("text", x = 3, y = 1.55, label = "c", color = "black", size = 5, fontface = "bold")




#Figure 2-----------------------------------------------------------------------
library(ggplot2)
library(readr)
library(dplyr)
library(lubridate)
library(data.table)
library(scales)
library(tidyr)

# Load and clean data
data <- read.table("Dataset S2.txt", header=T, dec="," ) %>%
  mutate(date_clean = dmy(date))

# Color palette
sample_levels <- unique(data$sample_name)
palette <- setNames(hue_pal()(length(sample_levels)), sample_levels)



# First appearance of larvae and prepupae per colony
event_labels_remove <- data %>%
  pivot_longer(cols = c(larvae, prepupae), names_to = "event", values_to = "value") %>%
  filter(value == 1) %>%
  group_by(sample_name, event) %>%
  arrange(date_clean) %>%
  slice_head(n = 1) %>%
  ungroup() %>%
  mutate(label = case_when(
    event == "larvae" ~ "First larvae",
    event == "prepupae" ~ "First prepupae"
  )) %>%
  group_by(date_clean, event) %>%
```

```
  mutate(y_offset = -10 + 40 * row_number()) %>%
  ungroup()

# First appearance of larvae per colony
event_labels_larvae <- data %>%
  pivot_longer(cols = c(larvae), names_to = "event", values_to = "value") %>%
  filter(value == 1) %>%
  group_by(sample_name, event) %>%
  arrange(date_clean) %>%
  slice_head(n = 1) %>%
  ungroup() %>%
  mutate(label = case_when(
    event == "larvae" ~ "First larvae",
  )) %>%
  group_by(date_clean, event) %>%
  mutate(y_offset = -10 + 40 * row_number()) %>%
  ungroup()

# First appearance of prepupae per colony
event_labels_prepupae <- data %>%
  pivot_longer(cols = c(prepupae), names_to = "event", values_to = "value") %>%
  filter(value == 1) %>%
  group_by(sample_name, event) %>%
  arrange(date_clean) %>%
  slice_head(n = 1) %>%
  ungroup() %>%
  mutate(label = case_when(
    event == "prepupae" ~ "First prepupae"
  )) %>%
  group_by(date_clean, event) %>%
  mutate(y_offset = 50 + 40 * row_number()) %>%
  ungroup()



# Mean and standard deviation for experimental group
mean_curve <- data %>%
  filter(sample_type == "experimental") %>%
  group_by(date_clean) %>%
  summarize(
    mean_concentration = mean(concentration_pg, na.rm = TRUE),
    sd = sd(concentration_pg, na.rm = TRUE),
    .groups = "drop"
  ) %>%
  mutate(
    sd_low = mean_concentration - sd,
    sd_high = mean_concentration + sd
  )

data$exp_day <- as.numeric(factor(data$date_clean, levels = sort(unique(data$date_clean))))
mean_curve$exp_day <- as.numeric(factor(mean_curve$date_clean, levels =
sort(unique(data$date_clean))))
event_labels_larvae$exp_day <- as.numeric(factor(event_labels_larvae$date_clean, levels =
sort(unique(data$date_clean))))
event_labels_prepupae$exp_day <- as.numeric(factor(event_labels_prepupae$date_clean, levels =
sort(unique(data$date_clean))))

#graph
ggplot(data, aes(x = exp_day, y = concentration_pg, color = sample_name)) +

  # SD ribbon
  geom_ribbon(
    data = mean_curve,
    aes(x = exp_day, ymin = sd_low, ymax = sd_high),
    fill = "grey60",
    alpha = 0.3,
    inherit.aes = FALSE
  ) +
```

```
  # annotation when larvae appear
  # arrows instead of text for larvae appearance
  geom_segment(
    data = event_labels_larvae,
    aes(x = exp_day, xend = exp_day,
        y = y_offset, yend = 0, color = sample_name),
    inherit.aes = FALSE,
    arrow = arrow(length = unit(0.2, "cm"), type = "closed"),
    size = 0.6,
    show.legend = FALSE
  )+
    geom_text(
    data = event_labels_larvae,
    aes(x = exp_day, y = y_offset + 0.2, label = label, color = sample_name),
    inherit.aes = FALSE,
    size = 3,
    show.legend = FALSE,
    angle = 45, hjust = 0.5
  )+


  # annotation when prepupae appear
  geom_segment(
    data = event_labels_prepupae,
    aes(x = exp_day, xend = exp_day,
        y = y_offset, yend = 0, color = sample_name),
    inherit.aes = FALSE,
    arrow = arrow(length = unit(0.2, "cm"), type = "closed"),
    size = 0.6,
    show.legend = FALSE
  )+
   geom_text(
    data = event_labels_prepupae,
    aes(x = exp_day, y = y_offset, label = label, color = sample_name),
    inherit.aes = FALSE,
    angle = 90,
    vjust = 0,
    size = 3,
    show.legend = FALSE
  ) +

  # Mean line
  geom_line(
    data = mean_curve,
    aes(x = exp_day, y = mean_concentration),
    color = "black",
    linewidth = 1.2,
    linetype = "dashed",
    inherit.aes = FALSE
  ) +

  # Individual data: Add `shape = sample_name` to geom_point
  geom_line(aes(group = sample_name), na.rm = TRUE, alpha = 0.3) +
  geom_point(aes(shape = sample_name), na.rm = TRUE, size = 2.5, alpha = 0.5) +  # Map shape to
sample_name

  # Color and shape scales
  scale_color_manual(values = palette) +
  scale_shape_manual(values = c(16, 16, 16, 17, 17)) +  # Assign distinct shapes (e.g., circle,
triangle, square, diamond)
  scale_fill_manual(values = palette) +

  # Show ticks every 5 days (starting at Day 1)
  scale_x_continuous(
    limits = c(2, max(data$exp_day)),
    breaks = seq(5, max(data$exp_day), by = 5),
    labels = seq(5, max(data$exp_day), by = 5)
  ) +
```

```
   labs(
    title = "Pheromone (Mean ± SD) according to larval development and foraging activity",
    x = "Day",
    y = "methyl 3-ethyl-2-hydroxy-4-methylpentanoate (MEHMP)",
    color = "amount (pg)"
  )+

theme_minimal() +
  theme(
    axis.line = element_blank(),
    axis.line.x.bottom = element_line(color = "black", size = 0.8),
    axis.line.y.left = element_line(color = "black", size = 0.8),
    axis.ticks = element_line(color = "black", size = 0.8),
    axis.ticks.length = unit(0.25, "cm"),
    axis.text.x = element_text(angle = 0, hjust = 0.5)
  )


#Figure 3 ------------------------------------------------------------------

#data
fig3=read.table("Dataset S3.txt", header=T, dec=".")


fig3$batch <- as.factor(fig3$batch)
fig3$treatment <- as.factor(fig3$treatment)
fig3$eggs <- as.numeric(fig3$eggs)
fig3$ants <- as.numeric(fig3$ants)

fig3_lastday=subset(fig3, day=="9")


#filtered data
fig3_lastday_filtered=subset(fig3_lastday, ID!="phero_pupae_1" & ID!="pupae_15") # remove
replicates where >33% of the ants died the last day

fig3_lastday_filtered1=subset(fig3_lastday_filtered, treatment !="precursor_pupae")
# count replicates per treatment
rep_counts_fig3 = fig3_lastday_filtered1 %>%
  group_by(treatment) %>%
  summarise(n = n())



#graph fig 3 last day

#with median and IQR
ggplot(fig3_lastday_filtered1, aes(x = factor(treatment, levels = c("Larvaecontact_pupae",
"phero_pupae", "larvaedistance_pupae", "pupae")), y = eggsperant)) +
  geom_jitter(aes(fill = treatment, color = "black"),
              height = 0, width = 0.2, size = 5, shape = 21, stroke = 0.5) +
  stat_summary(
    fun.data = function(x) {
      q <- quantile(x, probs = c(0.25, 0.75))
      m <- median(x)
      data.frame(y = m, ymin = q[1], ymax = q[2])
    },
    geom = "errorbar",
    width = 0,
    color = "black",
    size = 0.7
  ) +
  stat_summary(
    fun = median,
    geom = "point",
    shape = 18,
    size = 5,
    color = "black",
```

```
    fill = "white"
  ) +
  xlab("") +
  ylab("Number of eggs per worker") +
  theme(
    legend.position = "none",
    axis.title.x = element_text(size = 20),
    axis.text.x = element_text(size = 20, angle = 0, hjust = 0.5),
    axis.text.y = element_text(size = 18, angle = 0, hjust = 0.5),
    axis.title.y = element_text(size = 24),
    plot.title = element_text(hjust = 0.5, size = 25),
    panel.background = element_rect(fill = "white", color = NA),
    panel.grid.major = element_blank(),
    panel.grid.minor = element_blank(),
    plot.background = element_rect(fill = "white", color = NA),
    axis.line = element_line(color = "black", size = 0.5),
    plot.margin = margin(10, 10, 10, 10),
    panel.spacing = unit(0, "lines")
  ) +
  scale_y_continuous(limits = c(-0.1, 1.5), breaks = seq(0, 1.4, by = 0.2)) +
  scale_fill_manual(values = c(
    "Larvaecontact_pupae" = "#D7191C",
    "larvaedistance_pupae" = "#2E8A47",
    "phero_pupae" = "#8C51A5",
    "pupae" = "#2C7BB6"
  )) +
  scale_color_manual(values = c("black" = "black")) +
  scale_x_discrete(labels = c(
    "pupae" = "No
    larvae",
    "Larvaecontact_pupae" = "Larvae
    contact",
    "larvaedistance_pupae" = "Larvae
    distance",
    "phero_pupae" = "MEHMP"
  )) +
  geom_text(data = rep_counts_fig3, aes(x = treatment, y = -0.1, label = paste0("n = ", n)),
            inherit.aes = FALSE, size = 6)+
  annotate("text", x = 2, y = 1.2, label = "a", color = "black", size = 5, fontface = "bold")+
  annotate("text", x = 3, y = 1.2, label = "a", color = "black", size = 5, fontface = "bold")+
  annotate("text", x = 4, y = 1.45, label = "b", color = "black", size = 5, fontface = "bold")




#Supplementary Fig S2 - Concentration using TDU----------
concentration_TDU=c(0, 0, 0, 50, 50, 50, 100, 100, 100, 500, 500, 500, 1000, 1000, 1000)
area_TDU=c(0,0,0,2427,384,0,12562,11085,12453,77737,83664,64834,119816,159510,168282)

data_TDU <- data.frame(
  concentration = concentration_TDU,
  area = area_TDU
)

model <- lm(area_TDU ~  0+concentration_TDU)

# Print the model summary
summary(model)
#Adjusted R-squared:   0.979

ggplot(data_TDU, aes(x = concentration_TDU, y = (area_TDU/10000))) +
  geom_smooth(method = "lm", se = FALSE, color = "red", linetype = "solid", linewidth = 1) +
  geom_jitter(height = 0, width = 0, size = 2, shape = 19, stroke = 0.5, color = "black") +
  xlab("Amount (pg)") +
  ylab(expression(paste("Area under the peak (", 10^4, ")"))) +
  theme(
    legend.position = "none",
    axis.title.x = element_text(size = 16),
```

```
    axis.text.x = element_text(size = 14, angle = 0, hjust = 0.5),
    axis.text.y = element_text(size = 14, angle = 0, hjust = 0.5),
    axis.title.y = element_text(size = 16),
    plot.title = element_text(hjust = 0.5, size = 25),
    panel.background = element_rect(fill = "white", color = NA),
    panel.grid.major = element_blank(),
    panel.grid.minor = element_blank(),
    plot.background = element_rect(fill = "white", color = NA),
    axis.line = element_line(color = "black", size = 0.5),
    plot.margin = margin(10, 10, 10, 10),
    panel.spacing = unit(0, "lines")
  )+
  annotate("text", x = 500, y = 10, label = "R²=0.97", color = "black", size = 5, fontface =
"bold")+
  annotate("text", x = 500, y = 11, label = "y = 143.03x ", color = "black", size = 5, fontface
= "bold")


#Supplementary Fig S3 - Concentration using SPME----------------------
concentration_SPME=c(0, 0, 0, 50, 50, 50, 100, 100, 100, 250, 250,      250)
area_SPME=c(0, 0, 1001, 33210, 33753, 30984, 147078, 125822, 152215, 335535, 286285, 274804)

data_SPME <- data.frame(
  concentration = concentration_SPME,
  area = area_SPME
)

model <- lm(data_SPME$area ~  0+data_SPME$concentration)

# Print the model summary
summary(model)
#Adjusted R-squared:   0.98

ggplot(data_SPME, aes(x = concentration, y = (area/100000))) +
  geom_smooth(method = "lm", se = FALSE, color = "red", linetype = "solid", linewidth = 1) +
  geom_jitter(height = 0, width = 0, size = 2, shape = 19, stroke = 0.5, color = "black") +
  xlab("Amount (pg)") +
  ylab(expression(paste("Area under the peak (", 10^5, ")"))) +
  theme(
    legend.position = "none",
    axis.title.x = element_text(size = 16),
    axis.text.x = element_text(size = 14, angle = 0, hjust = 0.5),
    axis.text.y = element_text(size = 14, angle = 0, hjust = 0.5),
    axis.title.y = element_text(size = 16),
    plot.title = element_text(hjust = 0.5, size = 25),
    panel.background = element_rect(fill = "white", color = NA),
    panel.grid.major = element_blank(),
    panel.grid.minor = element_blank(),
    plot.background = element_rect(fill = "white", color = NA),
    axis.line = element_line(color = "black", size = 0.5),
    plot.margin = margin(10, 10, 10, 10),
    panel.spacing = unit(0, "lines")
  )+
  annotate("text", x = 125, y = 2, label = "R²=0.98", color = "black", size = 5, fontface =
"bold")+
  annotate("text", x = 125, y = 2.2, label = "y = 1206.95x", color = "black", size = 5,
fontface = "bold")



#Supplementary Fig S4 - MEMP-----------------------------------------------


#filtered data
fig3_lastday_filtered=subset(fig3_lastday, ID!="precursor_pupae_1" & ID!="pupae_15") # remove
replicates where >33% of the ants died the last day

# count replicates per treatment
rep_counts_fig3 = fig3_lastday_filtered %>%
```

```
  group_by(treatment) %>%
  summarise(n = n())

ggplot(fig3_lastday_filtered, aes(x = factor(treatment, levels = c("Larvaecontact_pupae",
"phero_pupae", "larvaedistance_pupae", "precursor_pupae", "pupae")), y = eggsperant)) +
  geom_jitter(aes(fill = treatment, color = "black"),
              height = 0, width = 0.2, size = 5, shape = 21, stroke = 0.5) +
  stat_summary(
    fun.data = function(x) {
      q <- quantile(x, probs = c(0.25, 0.75))
      m <- median(x)
      data.frame(y = m, ymin = q[1], ymax = q[2])
    },
    geom = "errorbar",
    width = 0,
    color = "black",
    size = 0.7
  ) +
  stat_summary(
    fun = median,
    geom = "point",
    shape = 18,
    size = 5,
    color = "black",
    fill = "white"
  ) +
  xlab("") +
  ylab("Number of eggs per worker") +
  theme(
    legend.position = "none",
    axis.title.x = element_text(size = 20),
    axis.text.x = element_text(size = 15, angle = 0, hjust = 0.5),
    axis.text.y = element_text(size = 18, angle = 0, hjust = 0.5),
    axis.title.y = element_text(size = 24),
    plot.title = element_text(hjust = 0.5, size = 25),
    panel.background = element_rect(fill = "white", color = NA),
    panel.grid.major = element_blank(),
    panel.grid.minor = element_blank(),
    plot.background = element_rect(fill = "white", color = NA),
    axis.line = element_line(color = "black", size = 0.5),
    plot.margin = margin(10, 10, 10, 10),
    panel.spacing = unit(0, "lines")
  ) +
  scale_y_continuous(limits = c(-0.1, 1.5), breaks = seq(0, 1.4, by = 0.2)) +
  scale_fill_manual(values = c(
    "Larvaecontact_pupae" = "#D7191C",
    "larvaedistance_pupae" = "#2E8A47",
    "phero_pupae" = "#8C51A5",
    "precursor_pupae" = "#d7bc19",
    "pupae" = "#2C7BB6"
  )) +
  scale_color_manual(values = c("black" = "black")) +
  scale_x_discrete(labels = c(
    "pupae" = "No
    larvae",
    "Larvaecontact_pupae" = "Larvae
    contact",
    "larvaedistance_pupae" = "Larvae
    distance",
    "precursor_pupae" = "MEMP",
    "phero_pupae" = "MEHMP"
  )) +
  geom_text(data = rep_counts_fig3, aes(x = treatment, y = -0.1, label = paste0("n = ", n)),
            inherit.aes = FALSE, size = 6)+
  annotate("text", x = 2, y = 1, label = "a", color = "black", size = 5, fontface = "bold")+
  annotate("text", x = 3, y = 1.2, label = "ab", color = "black", size = 5, fontface = "bold")+
  annotate("text", x = 4, y = 1.3, label = "bc", color = "black", size = 5, fontface = "bold")+
  annotate("text", x = 5, y = 1.45, label = "c", color = "black", size = 5, fontface = "bold")
```
